# Expression Levels of Detoxification Enzyme Genes from *Dendroctonus armandi* (Coleoptera: Curculionidae) Fed on a Solid Diet Containing Pine Phloem and Terpenoids

**DOI:** 10.3390/insects12100926

**Published:** 2021-10-11

**Authors:** Lulu Dai, Haiming Gao, Hui Chen

**Affiliations:** 1 Co-Innovation Center for Sustainable Forestry in Southern China, College of Forestry, Nanjing Forestry University, Nanjing 210000, China; dailulu@njfu.edu.cn; 2 College of Forestry, Northwest A&F University, Xianyang 712100, China; gaohaiming0996@163.com; 3 State Key Laboratory for Conservation and Utilization of Subtropical Agro-Bioresources, College of Forestry and Landscape Architecture, South China Agricultural University, Guangzhou 510642, China

**Keywords:** artificial feeding, chemical defense, Chinese white pine beetle, cytochrome P450, real-time qPCR

## Abstract

**Simple Summary:**

The bark beetle is the most well-known pest in coniferous trees worldwide. These insects only leave the host pine bark when they disperse to locate a new host. Determining how *Dendroctonus armandi* overcome the trees’ terpene-based defense systems has been the key problem in the study of bark beetles. Therefore, the aim of this study was to discover the molecular mechanism of insect detoxification enzymes’ ability to confer resistance to terpenes. For this purpose, the genes of cytochrome P450s, glutathione S-transferases, and carboxylesterases were studied in beetles given diets containing terpenes. The results suggest that beetles express different genes in response to terpenoids, and the responses of multiple detoxifying enzymes indicate these insects’ adaption to their chemical environment.

**Abstract:**

Bark beetles overcome the toxic terpenoids produced by pine trees by both detoxifying and converting them into a pheromone system. Detoxification enzymes such as cytochrome P450s, glutathione S-transferases, and carboxylesterases are involved in the ability of *Dendroctonus armandi* to adapt to its chemical environment. Ten genes from these three major classes of detoxification enzymes were selected to study how these enzymes help *D. armandi* to respond to the host defenses. The expression profile of these detoxification enzyme genes was observed in adult beetles after feeding on different types of diet. Significant differences were observed between two types of seminatural diet containing the phloem of pines, and a purely artificial diet containing five monoterpenes ((−)-α-pinene, (−)-β-pinene, (+)-3-carene, (±)-limonene, and turpentine oil) also caused differential transcript levels in the detoxification enzyme genes. The results suggest that monoterpenes enter the beetles through different routes (i.e., respiratory and digestive systems) and cause the expression of different genes in response, which might be involved in pheromone metabolism. In addition, the xenobiotic metabolism in bark beetles should be considered as a system comprising multiple detoxifying enzymes.

## 1. Introduction

The Chinese white pine beetle (*Dendroctonus armandi* Tsai and Li (Coleoptera: Curculionidae)) is an aggressive species that attacks healthy *Pinus armandii* Franch., which is the principal pine species in the Qinling Mountain Forest region [1,2,3]. Similarly, bark beetles only leave the host pine bark when they disperse to locate a new host. Bark beetles usually attack healthy trees with a disturbed terpenoid biosynthesis. Bark beetles must overcome the toxic terpenoids produced by the host pine during host colonization in order to maintain the beetle population [4]. During the infestation, the female beetles pioneer by boring through the healthy host and then attracting males with sex pheromones to colonize and reproduce [1]. There is likely a special metabolic mechanism in bark beetles to overcome the chemical defense of healthy Chinese white pine, allowing this tree to be their specific host selection and explaining the different behavior between the sexes. In our sampling location, insecticides had never been used for this forest pest, so the host allelochemical was the primary pressure for Chinese white pine beetle.

A diversity of terpenoids constitute the chemical defense of the host pine, including monoterpenes, sesquiterpenes, and diterpenes [5]. The induced resin not only delays the pioneer beetle’s invasion, but also reduces the attractiveness of the host [6]. Monoterpenes such as α-pinene, β-pinene, myrcene, limonene, and 3-carene can injure or kill insects through their toxic effects [7,8,9,10]. Additionally, monoterpenes (the volatile chemicals within terpenoids) have been considered to be involved with the host selection of some conifer bark beetles and to be precursors of beetle pheromones [11,12,13].

The insect resistance to insecticides conferred by the activity of three major detoxifying enzymes (cytochrome P450s (CYPs), glutathione S-transferases (GSTs), and carboxylesterase) has been studied for many years with the aim of revealing the microevolution and environmental adaptation of insects [14]. Multiple cytochrome P450 enzymes, which are derived from gene duplication and adaptive variation [15,16,17], are involved in the metabolism of exogenous and endogenous chemical compounds [18,19]. There is significant evidence indicating that bark beetles use multiple CYP enzymes to oxidize a broad range of toxic substrates from the host or environmental habitat [20,21,22,23]. GSTs are also multifunctional enzymes that conjugate insecticides with a glutathione moiety (GSH), and they often work in tandem with CYPs or other enzymes that aid in the detoxification, sequestration, or excretion of toxic compounds [24,25,26,27]. GSTs have previously been cloned from a budworm (*Choristoneura fumiferana* (Clem.) (Lepidoptera: Tortricidae)) that feeds on conifer tissues [28]. Esterases are frequently implicated in insect resistance to insecticides through gene amplification, upregulation, mutations, or a combination of these mechanisms in Hymenoptera, Lepidoptera, and Diptera species [14].

Using genome sequencing, many putative detoxifying enzyme genes have been identified in bark beetles, and some CYPs have been identified as bark-beetle-specific P450 genes, according to phylogenetic analysis with other species [29,30]. Differential expression levels of some detoxifying enzyme genes after feeding on host phloem have been discovered in both sexes [21]. In addition, the transcription levels of some P450 genes in unfed *Dendroctonus rhizophagus* Thomas and Bright (Coleoptera: Curculionidae) [31] and *Dendroctonus valens* LeConte (Coleoptera: Curculionidae) [32] individuals exposed to different monoterpenes have recently been studied. A transcriptome analysis of male and female *Dendroctonus ponderosae* Hopkins (Coleoptera: Curculionidae), either starved or fed in male-female pairs for 24 h on host tree tissues, demonstrated that beetle-specific P450, GST, and carboxylesterase were upregulated in insects after feeding on the host tissues [33]. In addition, the proteome profiles of mountain pine beetle fed on fresh host phloem showed the accumulation of several P450 and GST enzymes [34].

Previous research has indicated that some P450 genes in the CYP6 family were unique to bark beetles [10]. The genes, which code detoxifying enzymes, have special detoxification abilities in bark beetles’ invasion [10,35]. Within the distribution areas of the Chinese white pine beetle, there always some nonhost trees, *Pinus tabuliformis* Carr., growing in combination with host trees. The specific selection of this beetle for *P. armandii* as the host tree might be explained by the insects’ ability to detoxify different terpenes produced in these two *Pinus* species. Furthermore, the terpenoids which have been demonstrated to have toxic action in bark beetles in many previous studies [10] must influence the transcription of detoxifying enzyme genes through feeding behavior. Therefore, we investigated the transcription levels of 10 genes (4 cytochrome P450s, 4 glutathione S-transferases, and 2 carboxylesterase genes) in adult beetles after feeding on different solid diets which contained host pine (*P. armandii*) phloem powder, nonhost pine (*P. tabuliformis*) phloem powder, or terpenoids.

## 2. Materials and Methods

### 2.1. Insect Sample Preparation

The sample bark beetles were collected from infested host pine on the southern slope of the middle Qingling Mountains, Shaanxi, China (33°18′ N, 108°21′ E). Adults were sexed according to the shape of the seventh abdominal tergite, placed in plastic containers with moist paper, and taken back to the laboratory at 4 °C.

### 2.2. Insect Diet Preparation

Three solid insect diets were made for the Chinese white pine beetles: a purely artificial diet and two seminatural diets.

For the pure artificial diet we mixed yeast extract powder (10%), sucrose (10%), a multivitamin (a vitamin complex tablet with the following main components: vitamin A, C, D, E, B_1,2,6,12_, nicotinamide) (1.5%), wheat germ powder (15%), Wesson’s salt (0.5%), cellulose (60%), cholesterol (1.5%), potassium sorbate (1%), and methylparaben (0.5%) together into a paste with sterile water. All reagents using in artificial diet were bought from Sangong Biotech (Shanghai, China). After high-temperature (121 °C) sterilization, we dried it to 50% water content, and stored it at 4 °C in a sealed state.

For the seminatural diets, we prepared two diets containing phloem from the host tree (*Pinus armandii, Pa*) and from the nonhost tree (*Pinus tabuliformis, Pt*), respectively. The same ingredients as the pure artificial diet were mixed together and prepared as above, with the exception of cellulose, which was reduced to 10% by weight. The remaining 50% cellulose was replaced by the two types of pine phloem powders, respectively. Fresh phloem was collected from healthy pine and dried at 50 °C, and then ground to a powder using a plant tissue crusher.

### 2.3. Terpenoid Compounds Analysis by GC-MS

We analyzed the main terpenoids of the two pine phloem powders described above. One hundred milligrams of each type of pine phloem powder was added to 3000 μL of n-hexane (chromatographic grade, Sigma-Aldrich, St. Louis, MO, USA) in a 5-mL glass tube. The glass tube was vortexed for 30 s, placed in an ultrasonic oscillator for 15 min, and then left standing at 4 °C overnight. The extract was moved into a gas chromatography vial and stored at 4 °C.

Extracts (1 μL) were analyzed using a gas chromatograph/mass spectrometer (Thermo TRACE 1310ISQLT) (Thermo Scientific, Pittsburgh, PA, USA) equipped with a Varian VF-5MS (5% diphenyl–95% polymethylsiloxane) capillary column (30 m × 0.25 mm × 0.25 μm). Helium (>99%) was used as the carrier gas at a flow rate of 1 mL/min. The oven temperature program was set as 40 °C (hold 1 min), 3 °C/min to 130 °C (hold 1 min), 10 °C/min to 230 °C (hold 2 min), and 3 °C/min to 280 °C (hold 5 min). The quadrupole mass detector was operated in electron impact ionization mode at 70 eV, 150 °C. The ion source and transfer line temperature were set at 280 °C, and mass acquisition was ranged at 40–400 *m/z*. Turpentine oil used in this study was analyzed with the same method to confirm its main composition.

The phloem powder extracts and turpentine oil were each prepared in three tubes for biological repetition. The compound identification was based on comparisons of retention times and mass spectra with authentic standards, or with mass spectra in the National Institute of Standards and Technology libraries, NIST08 mass spectral database library (http://webbook.nist.gov/chemistry/) (accessed on 15 March 2021). The percentage compositions of each compound were computed from the GC peak areas.

### 2.4. Insects and Treatments for RT-qPCR

Male and female beetles were divided into seven groups, and each group contained 30 males and 30 females. One group was immediately killed using liquid nitrogen as time zero. The other six groups were placed individually into 1.5 mL centrifuge tubes with 1 g seminatural diet (three groups with *Pa* and another three groups with *Pt*). All tubes were kept in the dark at 20 °C for 24 h and 48 h to monitor the progression of feeding. Following feeding on the different diets, at the stated time points, all beetles were checked to determine their boring situation (length of gallery and frass). From each group, 12 beetles of each sex were chosen from each time point and diet type for use in realtime PCR analyses.

Another treatment for Chinese white pine beetles was performed with a purely artificial diet containing some terpenoids (added after autoclaving). According to terpenoid analysis of the host phloem, four monoterpenes ((−)-α-pinene (98%), (−)-β-pinene (99%), (+)-3-carene (90%), (±)-limonene (95%)), and turpentine oil (Aladdin Industrial Corporation, Shanghai, China) were selected and dissolved in DMSO (dimethylsulfoxide) (Aladdin Industrial Corporation, Shanghai, China) at two concentrations, 5% and 10% (*v*/*v*). We added 100 μL solution of each terpenoid at each concentration into 1.5 mL centrifuge tubes with 1 g pure artificial diet, and then placed one beetle in each tube. The concentrations of terpenoids in the pure artificial diet were higher than their levels in the natural phloem but were not high enough to kill the beetles, according to the beetles’ tolerance to terpenoids [10]. Thirty beetles of each sex were treated with one terpenoid at each concentration. Beetles fed on the purely artificial diet to which only 100 μL sterile water or DMSO was added were considered as the blank control and solvent control, respectively. Following feeding on the different diets for 24 h, all beetles were checked to determine their boring situation (length of gallery and frass). For each treatment group, 12 beetles of each sex were chosen from each diet containing different terpene concentrations for use in realtime PCR analyses.

### 2.5. RT-qPCR

The UNIQ-10 Column TRIzol Total RNA Isolation Kit (Sangong Biotech, Shanghai, China) was used to isolate total RNA from the treatment beetles according to the protocol. We checked the RNA integrity on 1% agarose gels and determined the quantity and purity using a NANO DROP 2000 (Thermo Scientific, Pittsburgh, PA, USA) by calculating the A260/A280. The TransScript One-Step gDNA Removal and cDNA Synthesis SuperMix (TransGen Biotech, Beijing, China) was used to synthesize cDNA using the total RNA as template.

We selected 10 genes from the three classes of Chinese white pine beetle detoxifying enzymes: 4 bark-beetle-specific cytochrome P450 genes from the CYP3 clade (accession numbers KR012828, KR012820, KR012847, and KR012845), 4 glutathione S-transferases from epsilon and sigma superfamilies, which are involved in the detoxification of xenobiotic compounds in beetles (accession numbers KJ637332, KP258218, and KP258220), and 2 carboxylesterases which are susceptible to terpenes (accession numbers MG676376 and MG676377) [10,35,36]. Based on their sequences, a pair of specific primers (Supplementary Material Table S1) were designed for each detoxifying enzyme gene using Primer Premier 5.0. A 20 µL reaction system was used with 0.4 μM of each primer and 10 µL qPCR premix (TransGen Biotech, Beijing, China). All samples were analyzed with the following conditions: predegeneration at 95 °C for 30 s, 40 cycles of degeneration at 95 °C for 5 s, renaturation at the TM of each pair of primers (Supplementary Material Table S1) for 15 s, and elongation at 72 °C for 20 s in a CFX96TM Real-Time PCR Detection System (Bio-Rad, Hercules, California, USA). Each treatment contained three biological replicates (three beetles for one biological replicate), and each biological replicate was performed three times as technical replicates.

A linear regression analysis was performed between the mean values of quantification cycles of different dilutions. We estimated the efficiency of each pair of primers and their PCR validation, as shown in Supplementary Material Table S1. Moreover, the specificity of each pair of primers was evaluated with a melting curve reaction.

Two genes, β-actin (KJ507199) and CYP4G55 (KR012821), were selected as the reference genes to normalize transcript levels of Chinese white pine beetle detoxifying enzyme genes, as they have been proven the most stable reference genes in similar studies [35,36,37]. Relative expressions of Chinese white pine detoxifying enzyme genes were determined using the Ct (∆∆CT) method [38]. All 2^−ΔΔCT^ values were (log_2_) transformed and subjected to one-way analysis of variance (ANOVA), and Tukey’s honestly significant difference test (HSD) was also used to compare treatment differences. SPSS 18.0 (IBM SPSS Statistics, Chicago, IL, USA) and SigmaPlot 12.0 software (Systat Software Inc., San Jose, CA, USA) were used for statistical analyses and plotting, respectively.

## 3. Results

### 3.1. Terpenoid Compounds of Seminatural Diet and Main Compositions of Turpentine Oil

**Figure 1:**
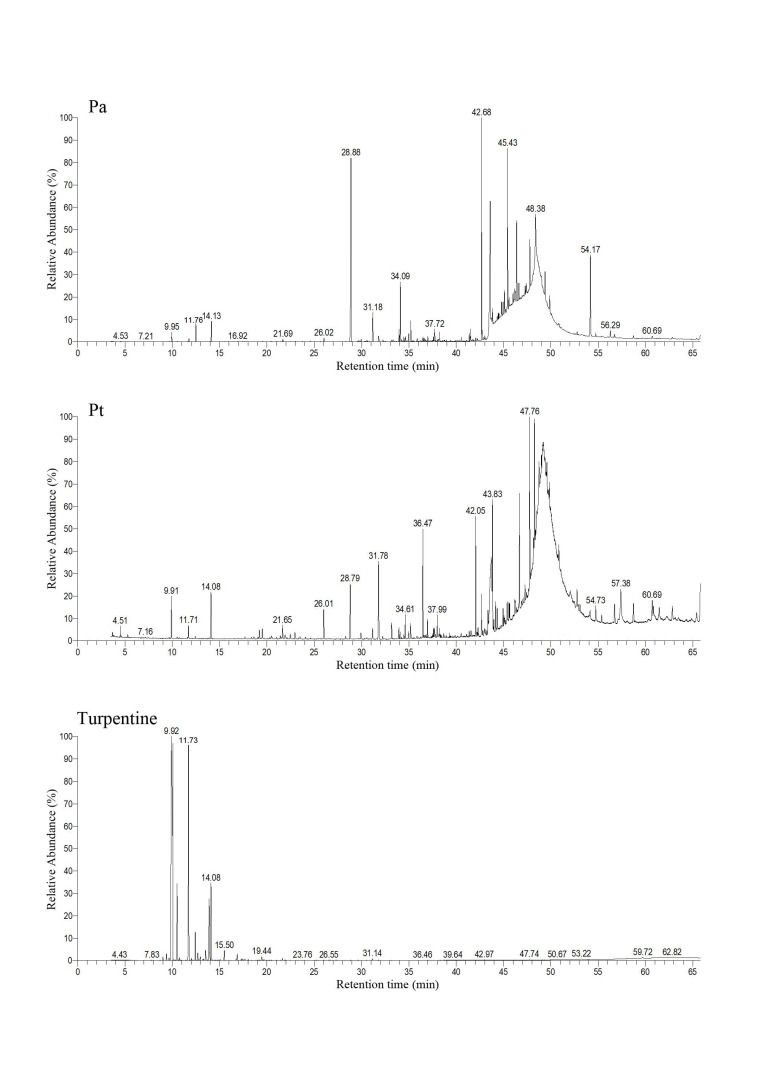
GC-MS chromatograms of *P. armandii* (*Pa*) and *P. tabuliformis* (*Pt*) phloem powders and the turpentine oil used in this research.

The turpentine used in this research consisted of five monoterpenes: α-pinene, camphene, β-pinene, β-myrcene, and D-limonene (Table 1). Among those compounds identified in pine bark powders, α-pinene, camphene, β-pinene, β-myrcene, and D-limonene were the main monoterpenes (Figure 1). However, the relative concentrations of these monoterpenes were under 10% (Table 1).

### 3.2. Detoxifying Enzyme Genes Expression in Beetles Feeding on Different Diets

#### 3.2.1. Feeding on Seminatural Diet

**Figure 2:**
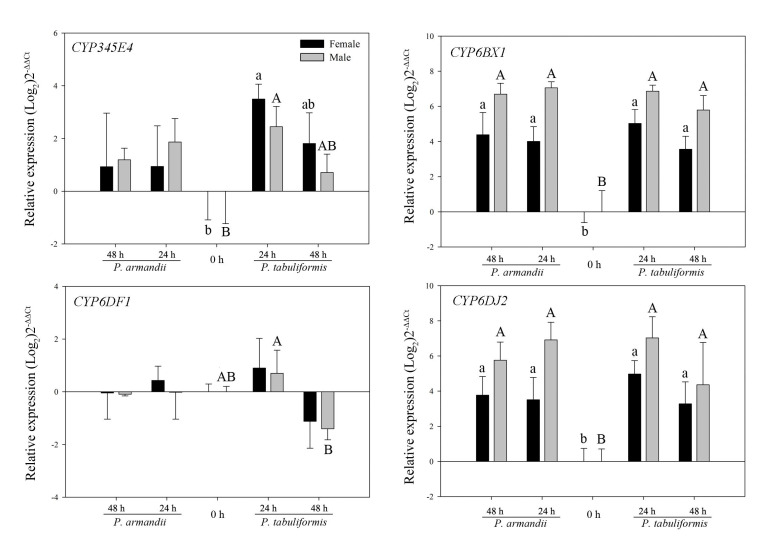
Realtime qPCR analysis of the relative expression of four P450 genes (mean ± SE) in *D. armandi* after being fed on two different diets for 24 and 48 h. Values > 1 on the logarithmic y-axis indicate upregulation. Gene expression was normalized with respect to the geometrical mean of β-actin and CYP4G55. The different letters indicate statistically significant differences in gene expression among different times for the same type of diet compared with Tukey’s HSD (*p* < 0.05). The 2^−ΔΔCt^ and SE values were log_2_ transformed for plotting.

After feeding on two types of seminatural diets, two P450 genes (CYP6BX1 and CYP6DJ2) were significantly upregulated in both sexes of the beetles after 24 and 48 h (Figure 2 and Table 2). Two additional genes, CYP345E4 and CYP6DF1, significantly responded only after feeding on the *Pt*-type seminatural diet in one or both sexes (Figure 2 and Table 2).

**Figure 3:**
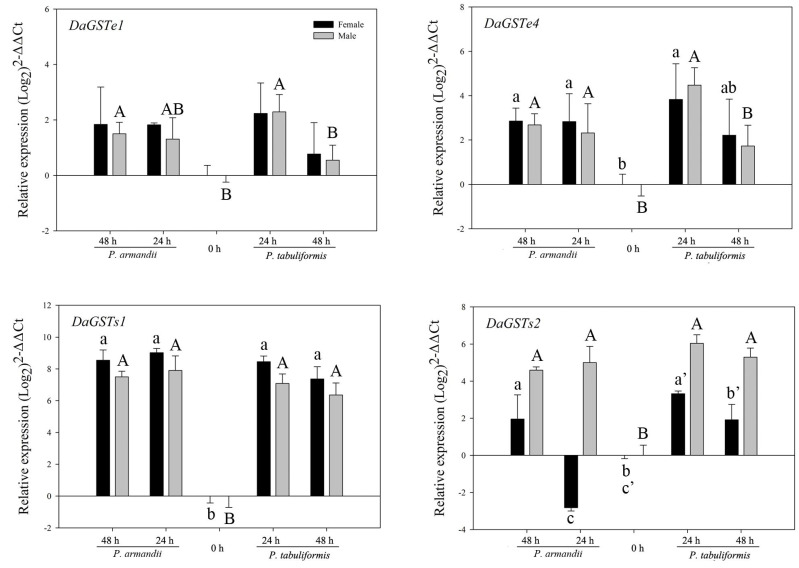
Realtime qPCR analysis of the relative expression of four GST genes (mean ± SE) in *D. armandi* after being fed on two different diets for 24 and 48 h. Values > 1 on the logarithmic y-axis indicate upregulation. Gene expression was normalized with respect to the geometrical mean of β-actin and CYP4G55. The different letters indicate statistically significant differences in gene expression among different times for the same diet, compared using Tukey’s HSD (*p* < 0.05). The 2^−ΔΔCt^ and SE values were log_2_ transformed for plotting.

Three GST genes (*DaGSTe4, DaGSTs1*, and *DaGSTs2*) were significantly upregulated in beetles of both sexes after feeding on the seminatural diets (*Pa* or *Pt*), with the exception of *DaGSTs2*, which was downregulated in females after feeding on the *Pa*-type seminatural diet for 24 h (Figure 3 and Table 2). In addition, the transcription levels of *DaGSTe1* were significantly upregulated only in males fed on *Pt* and *Pa* (Figure 3 and Table 2).

**Figure 4:**
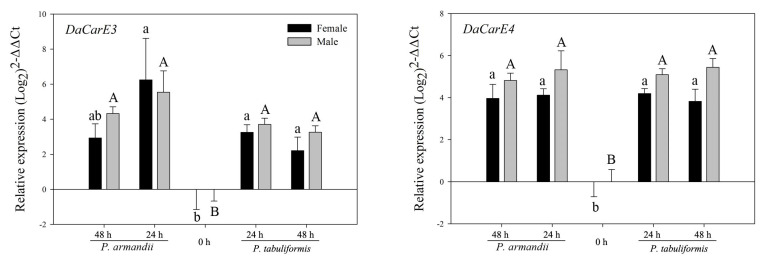
Realtime qPCR analysis of the relative expression of two CarE genes (mean ± SE) in *D. armandi* after being fed on two different diets for 24 and 48 h. Values > 1 on the logarithmic y-axis indicate upregulation. Gene expression was normalized with respect to the geometrical mean of β-actin and CYP4G55. The different letters indicate statistically significant differences in gene expression among different times for the same diet, compared using Tukey’s HSD (*p* < 0.05). The 2^−ΔΔCt^ and SE values were log_2_ transformed for plotting.

Two CarE genes (*DaCarE3* and *DaCarE4*) were significantly upregulated in beetles of both sexes after feeding on seminatural diets (*Pa* or *Pt*) (Figure 4 and Table 2).

#### 3.2.2. Feeding on Pure Artificial Diet Containing Different Terpenoids

No significant differences (*p* > 0.05) were found in the transcription levels between the blank and solvent controls for most of these 10 genes using the independent sample *t*-test (Table 3). Compared with beetles that consumed pure artificial diet as blank and solvent controls, most genes of the three major classes of detoxification enzymes were significantly downregulated in both sexes (CYP345E4, CYP6DF1, *DaGSTe1, DaGSTe4, DaGSTs1, DaGSTs2, DaCarE3,* and *DaCarE4*) after feeding on pure artificial diet containing (−)-α-pinene, (−)-β-pinene, and (+)-3-carene (Table 3). However, the transcription levels of these eight genes in beetles fed on pure artificial diet containing limonene and turpentine varied and were different from those of the other three monoterpenes.

**Figure 5:**
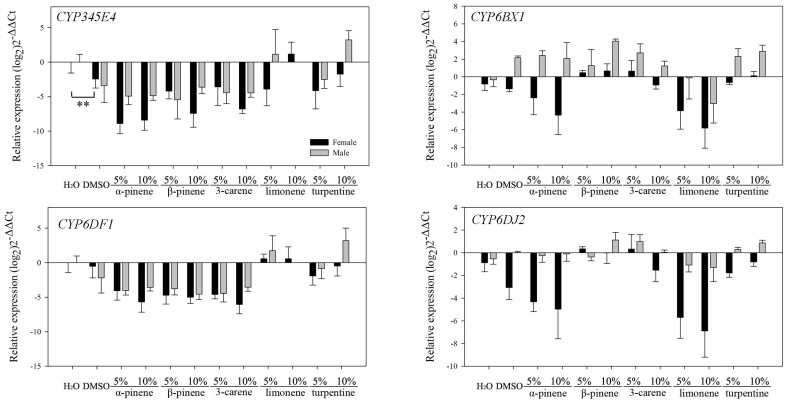
Realtime qPCR analysis of four P450 genes relative expression (mean ± SE) in *D. armandi* after fed on pure artificial feed with stimuli at different concentrations for 24 h. Values > 1 on the logarithmic y-axis indicate upregulation. Gene expression was normalized with respect to the geometrical mean of β-actin and CYP4G55. Asterisks indicate significant differences between the blank control and the solvent control based on the independent sample *t*-test of each gene’s expression (** *p* < 0.01). Statistically significant differences in gene expression among concentrations are shown in Table 3. The 2^−ΔΔCt^ and SE values were log_2_ transformed for plotting.

CYP345E4: In males, this gene was downregulated after feeding on pure artificial diet containing a low concentration (5%) of turpentine but upregulated for the diet containing a high concentration (10%) of turpentine (Figure 5 and Table 3). The same trend observed in the males feeding on diets containing turpentine appeared in the females feeding on diets containing limonene (Figure 5 and Table 3).

CYP6DF1: This gene was upregulated only in males after feeding on pure artificial diet containing a high concentration (10%) of turpentine (Figure 5 and Table 3).

CYP6BX1: This gene was significantly downregulated in females that were exposed to (−)-α-pinene and limonene at low and high concentrations compared with the blank and solvent controls (Figure 5 and Table 3). However, (−)-β-pinene and turpentine at a high concentration (10%) could induce this downregulation in males (Figure 5 and Table 3).

CYP6DJ2: This gene’s expression levels in females were similar to those of CYP6BX1, as (−)-α-pinene and limonene inhibited expression in females (Figure 5 and Table 3).

**Figure 6:**
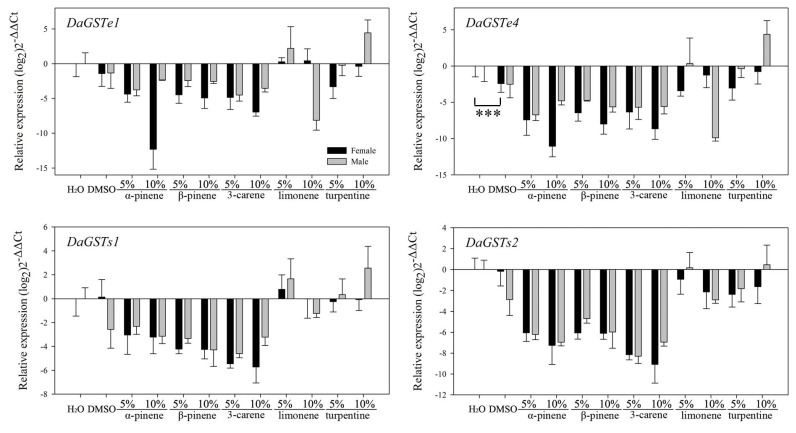
Realtime qPCR analysis of four GST genes relative expression (mean ± SE) in *D. armandi* after fed on pure artificial diet with stimuli at different concentrations for 24 h. Values > 1 on the logarithmic y-axis indicate upregulation. Gene expression was normalized with respect to the geometrical mean of β-actin and CYP4G55. Asterisks indicate significant differences between the blank control and the solvent control based on the independent sample *t*-test of each gene’s expression (*** *p* < 0.001). Statistically significant differences in gene expression among concentrations are shown in Table 3. The 2^−ΔΔCt^ and SE values were log_2_ transformed for plotting.

*DaGSTe1, DaGSTe4*: In males, these genes were significantly upregulated after feeding on a purely artificial diet containing a high concentration of turpentine (10%), but they were downregulated after feeding on diet containing a high concentration of limonene (10%) (Figure 6 and Table 3).

*DaGSTs1*, *DaGSTs2*: These two genes were overexpressed in males after feeding on a diet containing a low concentration of limonene (5%) (Figure 6 and Table 3). In addition, turpentine had no effect on *DaGSTs2*.

**Figure 7:**
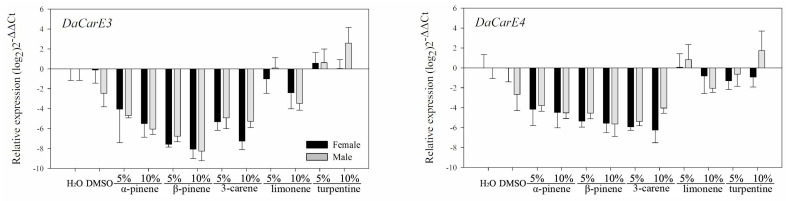
Realtime qPCR analysis of the relative expression of two CarE genes (mean ± SE) in *D. armandi* after fed on a purely artificial diet with stimuli at different concentrations for 24 h. Values > 1 on the logarithmic y-axis indicate upregulation. Gene expression was normalized with respect to the geometrical mean of β-actin and CYP4G55. Statistically significant differences in gene expression among concentrations are shown in Table 3. The 2^−ΔΔCt^ and SE values were log_2_ transformed for plotting.

*DaCarE3*, *DaCarE4*: In males, these two genes were upregulated after feeding on a purely artificial diet containing a high concentration of turpentine (10%) but downregulated after feeding on a diet containing limonene (Figure 7 and Table 3).

## 4. Discussion

In this study, we evaluated the expression of detoxifying enzyme genes in *Dendroctonus* species, induced by diets containing host tree phloem. Our results suggest a contribution of these enzymes to overcoming the host defenses [21,33]. Our previous research showed that a period of feeding on host bark induced rapid and dramatic changes in the gene transcription levels of the three major classes of detoxifying enzymes in both sexes [10,35,36]. The specific response of P450 genes after feeding on host bark has been reported in other bark beetle species, such as *Ips pini* [39], *I. paraconfusus* [21], and *D. ponderosae* [33,40]. This differential expression of CYP6BX1, CYP6DJ2, *DaGSTe4, DaGSTs1*, and *DaCarE3* indicates that they have some relationship with the detoxification of terpenoids [18] or with the transition from terpenoids to pheromones [41,42,43]. In addition, the special influence of the *Pt*-type seminatural diet on the transcription levels of some P450, GST, and carboxylesterase genes in beetles might provide some explanation for their specific selection of *P. armandii* as a host tree [1] (*P. armandii* has a lower content of limonene than *P. tabuliformis*, and limonene has a higher lethality for *D. armandi* than α-pinene and β-pinene) [10]. This assertion is strengthened by the fact that *P. tabuliformis* always grows together with *P. armandii* within the distribution areas of the bark beetles.

A higher concentration of monoterpenes in conifers caused by bark beetle invasion [15,44] could result in a higher mortality of bark beetles [9,10,13,44]. The variety of genes and proteins related to detoxification and physiological stress in insects have been proven to be related to the tolerance of plant-produced toxins [4]. Additionally, differential expression patterns of detoxifying enzymes between sexes have been observed after exposure to host volatiles in several bark beetle species, and changes in their transcripts in the same species were shown to be overexpressed in response to terpenoids from conifer hosts [31,32,33]. Furthermore, after exposure to host phloem for 24 h, the transcripts for P450s, GSTs, and esterases showed significant changes, which could play a role in the detoxification of host defense compounds and promote beetles’ development and reproduction [4]. The identification and expression of the P450, GST, and carboxylesterase genes in *D. armandi* suggests that many detoxifying enzymes might be induced or inhibited by monoterpenes from host tissues, regardless of whether they are administered alone or in combination, having an effect on insect positive or negative taxis [10,35].

However, bark beetles must metabolize these monoterpenes as precursors for the biosynthesis of kairomones or pheromones to enable chemical communication [13]. As the volatile monoterpenes are exuded by damaged phloem, they can enter bark beetles’ bodies via three pathways: the respiratory system, digestive tract, and cuticle [45].

In contrast to previously used methods, in this study, we added the host chemical compounds into a purely artificial diet to feed the beetles. This method was used to simulate the terpenoids’ effects on beetles when they bore galleries under host bark. The results showed that three monoterpenes, (−)-α-pinene, (−)-β-pinene, and (+)-3-carene, had an effect on most detoxifying enzyme genes in both sexes, leading to downregulated transcription levels. However, some detoxifying enzyme genes, such as CYP6BX1 and CYP6DJ2, were induced by (−)-β-pinene and (+)-3-carene in one sex. Similarly, the gene transcripts encoding for cytochrome P450s from *D. ponderosae* adults during early host colonization showed significant and varied regulations in transcription levels for beetles of both sexes [4].

Previous research indicates that (−)-α-pinene constitutes the highest proportion of the volatile monoterpenes in the resin of *P. armandii* [5], and the concentration of limonene in the resin of *P. armandii* was found to increase after attack by the beetles in [46]. α-Pinene might be the most important monoterpene for the innate resistance of the host, and limonene for the induced resistance, since the primary components of turpentine determined in this study were α-pinene (56.62%), β-pinene (18.34%), and limonene (11.73%). This could explain why we consistently observed a pronounced concentration-dependent effect of limonene and turpentine on the expression of the three classes of detoxifying enzymes in males. Limonene always results in higher mortality for male beetles in toxicity tests when compared to other terpenoids at lower concentrations [10].

Without knowing the substrates/products of these enzymes, we cannot determine the actual role of these genes in detoxification or endogenous hormone metabolism. The expression of genes coding for functional cytochrome P450s in bark beetles is modified upon feeding on host tissues [20,21,40], suggesting a potential role in metabolic detoxification [33]. Similarly, glutathione-S-transferases and esterases alter the solubility of xenobiotics in the insect body to facilitate their excretion, and they function in concert with cytochrome P450s, with a proven role in detoxification [14,27,28].

Our group has studied the expression profiles of ten genes from these three classes of detoxifying enzymes in Chinese white pine beetles after feeding on host phloem or exposure to monoterpenes [10,35,36]. However, some variations in the expression of these genes were found after feeding on two types of seminatural diets and purely artificial diets that contained different concentrations of monoterpenes. The two seminatural diets induced the expression of substantially more detoxifying enzyme genes than when the host phloem was used directly as the diet. The addition of monoterpenes to the diet had more negative effects on cytochrome P450s and GSTs than was observed in exposure experiments [10,35]. Therefore, the entry of monoterpenes into the beetles through different routes (respiratory and digestive systems) causes a differential gene expression response, which might be involved in putative detoxification [27,28,33] or hormone metabolism [21].

The results of this study suggest some relationship between bark beetle detoxifying enzyme genes and the beetles’ adaptation with their host tree. In addition, the primary functional characteristic of the detoxifying enzymes is diversification in xenobiotic metabolism. However, for bark beetles, the primary functions of the detoxifying enzymes involved in their specific colonization behavior and adaptation within their special environment merit additional research.

## Figures and Tables

**Table 1:** The terpenoid compounds’ relative abundance in the bark powders of *P. armandii* (*Pa*) and *P. tabuliformis* (*Pt*), and the main constituents of the turpentine used in this paper.

Compound	Molecular Weight	*Pa*		*Pt*		Turpentine	
		RT (min)	RA (%)	RT (min)	RA (%)	RT (min)	RA (%)
α-pinene	136	9.95	0.71 ± 0.22	9.91	1.87 ± 1.14	9.92	56.62 ± 2.35
camphene	136	10.55	0.04 ± 0.01	10.51	0.08 ± 0.05	10.50	5.20 ± 0.47
β-pinene	136	11.76	0.26 ± 0.08	11.71	0.76 ± 0.35	11.73	18.34 ± 1.16
β-myrcene	136	12.48	1.37 ± 0.42	12.44	0.15 ± 0.06	12.43	1.89 ± 0.04
D-limonene	136	14.13	1.67 ± 0.52	14.08	2.68 ± 0.97	14.08	11.73 ± 0.50
trans-verbenol	152	19.54	0.04 ± 0.01	19.51	1.34 ± 0.91		
bornyl acetate	196	26.02	0.36 ± 0.09	26.01	2.05 ± 0.53		
longifolene	204	31.18	2.72 ± 0.70	31.15	0.80 ± 0.19		
caryophyllene	204	31.80	0.54 ± 0.15	31.97	5.98 ± 1.36		
humulene	204	33.18	0.13 ± 0.04	33.17	1.04 ± 0.22		
germacrene D	204	34.09	4.78 ± 1.32	34.09	0.37 ± 0.08		

RT: retention time; RA: relative abundance.

**Table 2:** One-way ANOVA and Tukey test results regarding the expression of *D*. *armandi* detoxifying enzyme genes after being fed seminatural diets.

Gene	Sex	*df*	*Pa*		*Pt*	
			*F*	*p*-Value	*F*	*p*-Value
CYP345E4	♀	2	0.342	0.723	9.645	**0.013**
	♂	2	3.231	0.112	5.478	**0.044**
CYP6BX1	♀	2	20.240	**0.002**	38.246	**<0.001**
	♂	2	72.225	**<0.001**	54.293	**<0.001**
CYP6DF1	♀	2	0.463	0.650	3.834	0.085
	♂	2	0.020	0.980	10.552	**0.011**
CYP6DJ2	♀	2	12.220	**0.008**	21.352	**0.002**
	♂	2	47.810	**<0.001**	14.624	**0.005**
*DaGSTe1*	♀	2	5.106	0.051	4.409	0.066
	♂	2	7.311	**0.025**	17.312	**0.003**
*DaGSTe4*	♀	2	11.300	**0.009**	6.098	**0.036**
	♂	2	8.392	**0.018**	25.823	**0.001**
*DaGSTs1*	♀	2	348.950	**<0.001**	212.283	**<0.001**
	♂	2	121.334	**<0.001**	96.182	**<0.001**
*DaGSTs2*	♀	2	29.396	**0.001**	33.313	**0.001**
	♂	2	63.084	**<0.001**	128.045	**<0.001**
*DaCarE3*	♀	2	11.536	**0.009**	11.718	**0.008**
	♂	2	36.506	**<0.001**	51.775	**<0.001**
*DaCarE4*	♀	2	46.650	**<0.001**	53.767	**<0.001**
	♂	2	60.536	**<0.001**	140.578	**<0.001**

Bold fonts indicate significant differences among different elapsed feeding times for the same type of feed in the same sex as determined by one-way ANOVA (*p* < 0.05).

**Table 3:** One-way ANOVA analysis of detoxifying enzyme gene expression from *D*. *armandi* with each stimulus at different concentrations.

Gene	Sex	Independent Sample *t*-Test *		*df*	(−)-α-Pinene		(−)-β-Pinene		(+)-3-Carene		Limonene		Turpentine	
		t	*p*-Value		*F*	*p*-Value	*F*	*p*-Value	*F*	*p*-Value	*F*	*p*-Value	*F*	*p*-Value
CYP345E4	♀	5.961	**0.004**	3	27.185	**<0** **.001**	12.239	**0** **.002**	8.006	**0** **.009**	4.879	**0.032**	2.432	0.140
	♂	−0.855	0.441	3	6.963	**0** **.013**	3.917	0.054	5.303	**0** **.026**	2.508	0.162	10.012	**0.004**
CYP6DF1	♀	0.961	0.391	3	10.252	**0.004**	11.567	**0** **.003**	14.903	**0.001**	0.392	0.762	0.941	0.465
	♂	−1.060	0.349	3	6.223	**0** **.017**	6.844	**0** **.013**	5.959	**0.019**	3.372	0.104	5.660	**0.022**
CYP6BX1	♀	1.999	0.116	3	36.981	**<0** **.001**	2.167	0.170	5.126	**0.029**	30.365	**<0** **.001**	1.897	0.209
	♂	−0.900	0.419	3	1.463	0.296	1.098	0.404	1.512	0.284	7.713	**0.010**	1.402	0.311
CYP6DJ2	♀	2.671	0.056	3	13.315	**0.002**	2.620	0.123	3.670	0.063	60.936	**<0** **.001**	4.630	**0.037**
	♂	0.082	0.939	3	0.788	0.533	0.276	0.841	0.954	0.460	3.138	0.087	0.449	0.725
*DaGSTe1*	♀	1.945	0.124	3	22.199	**<0.001**	6.418	**0.016**	11.909	**0.003**	0.844	0.507	2.254	0.159
	♂	−0.848	0.444	3	3.717	0.061	2.078	0.182	5.994	**0.019**	12.329	**0.002**	5.949	**0.020**
*DaGSTe4*	♀	19.274	**<0.001**	3	28.401	**<0.001**	23.347	**<0** **.001**	16.017	**0.001**	3.632	0.064	2.564	0.128
	♂	−0.042	0.969	3	11.259	**0.003**	8.832	**0.006**	7.480	**0.010**	13.152	**0.002**	7.589	**0.010**
*DaGSTs1*	♀	−0.584	0.591	3	4.662	**0.036**	14.691	**0.001**	20.552	**<0.001**	0.202	0.892	0.049	0.985
	♂	0.088	0.934	3	5.637	**0.023**	7.591	**0.010**	11.543	**0.003**	6.311	**0.017**	6.383	**0.016**
*DaGSTs2*	♀	0.643	0.555	3	24.539	**<0.001**	37.575	**<0** **.001**	43.895	**<0.001**	1.463	0.296	2.180	0.168
	♂	1.701	0.164	3	35.148	**<0.001**	14.041	**0.001**	45.974	**<0.001**	6.591	**0.015**	3.519	0.069
*DaCarE3*	♀	0.817	0.460	3	5.661	**0.022**	59.159	**<0** **.001**	35.633	**<0** **.001**	1.871	0.213	0.221	0.879
	♂	−0.463	0.668	3	23.826	**<0** **.001**	39.519	**<0** **.001**	15.217	**0.001**	8.075	**0.008**	6.862	**0.013**
*DaCarE4*	♀	0.686	0.530	3	8.508	**0.007**	23.794	**<0** **.001**	26.736	**<0** **.001**	0.231	0.872	0.926	0.471
	♂	2.293	0.084	3	10.537	**0.004**	12.757	**0.002**	14.858	**0.001**	5.243	**0.027**	4.358	**0.043**

Bold fonts indicate significant differences among water, DMSO, and different concentrations (5% and 10%) of the same stimulus in the same sex with one-way ANOVA (α = 0.05). * Independent sample *t*-test of each gene’s expression between the blank control and the solvent control.

## Supplementary Materials

The following are available online at https://www.mdpi.com/article/10.3390/insects12100926/s1. Supplementary Material Table S1: Primers used for RT-qPCR.
