# Supplementary material for: Expression Levels of Detoxification Enzyme Genes from Dendroctonus armandi (Coleoptera: Curculionidae) Fed on a Solid Diet Containing Pine Phloem and Terpenoids
Source: Insects. 2021 Oct 11;12(10):926. doi: 10.3390/insects12100926 (PMC8541301; doi:10.3390/insects12100926)
Supplement: Supplementary file 1 [file insects-12-00926-s001.zip › insects-1380890-supplementary.pdf]

Supplementary material Table S1. Primers used for RT-qPCR.

Gene	Accession No.	Sequence (5′ to 3′)	Efficiency (%)	TM (°C)	Amplicon length [illegible]
CYP345E4	KR012828	F-TTATTGAAGAAAGCCAGGACA<br>R-CACGGACGCAAAGTTCATACA	104.7	61	141
CYP6BX1	KR012820	F-TGACGGCATTCTTTCTTAT<br>R-CATCACTTCCTGGTACTACAT	96.1	60	140
CYP6DF1	KR012847	F-GGAGGCGTCTACTGGGCGTTTA<br>R-CGCCACTGCTCGCCAGGTAA	92.0	59	167
CYP6DJ2	KR012845	F-AAATGTGACCCGTGTATGTG<br>R-TTCGGACTAAATCGTTCTGG	93.2	61	159
DaGSTe1	KJ637332	F-TTTGGGCTTGGATGTAGAAT<br>R-TTGAAGTGTTGGAACCGTGT	103.9	60	106
DaGSTe4	KP258220	F-TGATGACGGCTCGAATA<br>R-GCATGACTGTCCCAAAT	98.4	60	154
DaGSTs1	KP258218	F-TAATGAAGGAACCAGACCCAA<br>R-GTCCAATAGCACGGCAAAGA	94.5	60	170
DaGSTs2	KP258219	F-GGAACTACTCCTGCTGGTCAAC<br>R-CGTATCAACGGCGGCATCAAT	96.8	53	153
DaCarE3	MG676376	F-AGATGCAGCAGAGGACAGTCACATG<br>R-GCTCCATTACGAAGACCGCCACCAT	94.7	60	171
DaCarE4	MG676377	F-GGCAATATTCGTATGCAGCGGCGTT<br>R-CCAGGTCGTTCGGCTTCGTGAGTT	103.8	66	144
β-actin	KJ507199	F-TGTTGCGGCTCTTGTCGTTGA<br>R-ACCCATACCGACCATCACTCC	93.4	60	133

F= Forward primer, R= Reverse primer
